# Exploration of Fuzheng Yugan Mixture on COVID-19 based on network pharmacology and molecular docking

**DOI:** 10.1097/MD.0000000000032693

**Published:** 2023-01-20

**Authors:** Xinyu Jiang, Jie Zhou, Zhongming Yu, Xueya Gu, Ying Lu, Yanmin Ruan, Tianyue Wang

**Affiliations:** a The First Clinical Medical College, Zhejiang Chinese Medical University, Hangzhou, China; b Department of Physiology, Zhejiang Chinese Medical University, Hangzhou, China; c Center for Medicinal Resources Research, Tongde Hospital of Zhejiang Province, Hangzhou, China; d Central Preparation Room, Tongde Hospital of Zhejiang Province, Hangzhou, China; e The Second Clinical Medical College, Zhejiang Chinese Medical University, Hangzhou, China.

**Keywords:** COVID-19, Fuzheng Yugan mixture, molecular docking, network pharmacology, traditional Chinese medicines

## Abstract

After the World Health Organization declared coronavirus disease 2019 (COVID-19), as a global pandemic, global health workers have been facing an unprecedented and severe challenge. Currently, a mixturetion to inhibit the exacerbation of pulmonary inflammation caused by COVID-19, Fuzheng Yugan Mixture (FZYGM), has been approved for medical institution mixturetion notification. However, the mechanism of FZYGM remains poorly defined. This study aimed to elucidate the molecular and related physiological pathways of FZYGM as a potential therapeutic agent for COVID-19. Active molecules of FZYGM were obtained from the Traditional Chinese Medicine Systems Pharmacology Database and Analysis Platform (TCMSP), while potential target genes of COVID-19 were identified by DrugBank and GeneCards. Compound-target networks and protein-protein interactions (PPI) were established by Cytoscape_v3.8.2 and String databases, respectively. The gene ontology (GO) analysis and Kyoto Encyclopedia of Genes and Genomes (KEGG) pathway enrichment analysis were performed. Finally, a more in-depth study was performed using molecular docking. Our study identified 7 active compounds and 3 corresponding core targets. The main potentially acting signaling pathways include the interleukin (IL)-17 signaling pathway, tumor necrosis factor (TNF) signaling pathway, Toll-like receptor signaling pathway, Th17 cell differentiation, and coronavirus disease-COVID-19. This study shows that FZYGM can exhibit anti-COVID-19 effects through multiple targets and pathways. Therefore, FZYGM can be considered a drug candidate for the treatment of COVID-19, and it provides good theoretical support for subsequent experiments and clinical applications of COVID-19.

## 1. Introduction

The coronavirus disease 2019 (COVID-19) pandemic is caused by the rapid international spread of a coronavirus called SARS-CoV-2.^[1]^ COVID-19 has a relatively long incubation period and highly contagious. Up to Octuber 22, 2021, more than 4.9 million fatalities and 242.3 million infections have been recorded, making it one of the most widespread pandemics in history.^[2]^ The main clinical manifestations of COVID-19 are including fever, cough, respiratory distress, severe gastrointestinal distress and acute respiratory distress.^[3]^ The consequences of COVID-19 also severely affect the prognosis of patients.^[4]^ In China, the spread of the epidemic has been effectively controlled and effective treatment has been carried out for patients with COVID-19. Among them, traditional Chinese medicine (TCM) has made an important contribution to the prevention and control of the epidemic, such as anti-inflammatory, improving immunity, and stimulating their own resistance to disease and recovery ability.

Fuzheng Yugan Mixture (FZYGM) is a hospital preparation of Tongde Hospital of Zhejiang Province. This mixture has now been passed by Zhejiang Provincial Drug Administration and was approved for medical institution preparation filing (NO. Z20200043001) for the prevention and control of pneumonia epidemic with COVID-19 infection. According to the theory of Chinese medicine, “the body is full of positive energy, so that the pathogenic agents hardly invade the body.” To prevent influenza and pneumonia, it is advisable to start with benefiting the Qi and strengthening the body. This mixture is based on the classical mixture Yu Ping Feng San and composed of 7 herbs, namely *Hedysarum Multijugum Maxim, Saposhnikoviae Radix, Atractylodes Macrocephala Koidz, licorice, Lonicerae Japonicae Flos, Pogostemon Cablin (Blanco) Benth and Perilla Frutescens*.

*Hedysarum Multijugum Maxim* possesses detoxifying properties and exhibits bioactivities such as immunomodulatory and anti-inflammatory effects.^[5]^ *Saposhnikoviae Radix* has been used for the treatment of common cold and cough, which demonstrated antimicrobial, antiviral and antioxidant activities.^[6]^ *Atractylodes Macrocephala Koidz* is a strengthening spleen drug for eliminating phlegm and dampness in China.^[7]^ *licorice* has been used to relieve coughing and alleviate pain since ancient times.^[8]^ *Lonicerae Japonicae Flos* is a commonly used medicine to treat phyma, sore and fever.^[9]^ *Pogostemon Cablin (Blanco) Benth* and *Perilla Frutescens* have been used for respiratory symptoms, such as asthma, cold, fever, and stuffy nose.^[10,11]^ Among them, Perilla Frutescens also has been used for some intestinal disorders.^[11]^ However, there are no comprehensive analysis of pharmacological mechanisms about this mixture. Therefore, the objective of this study is to provide essential functional chemical compounds and associated physiological pathways of FZYGM.

**Figure 1.**
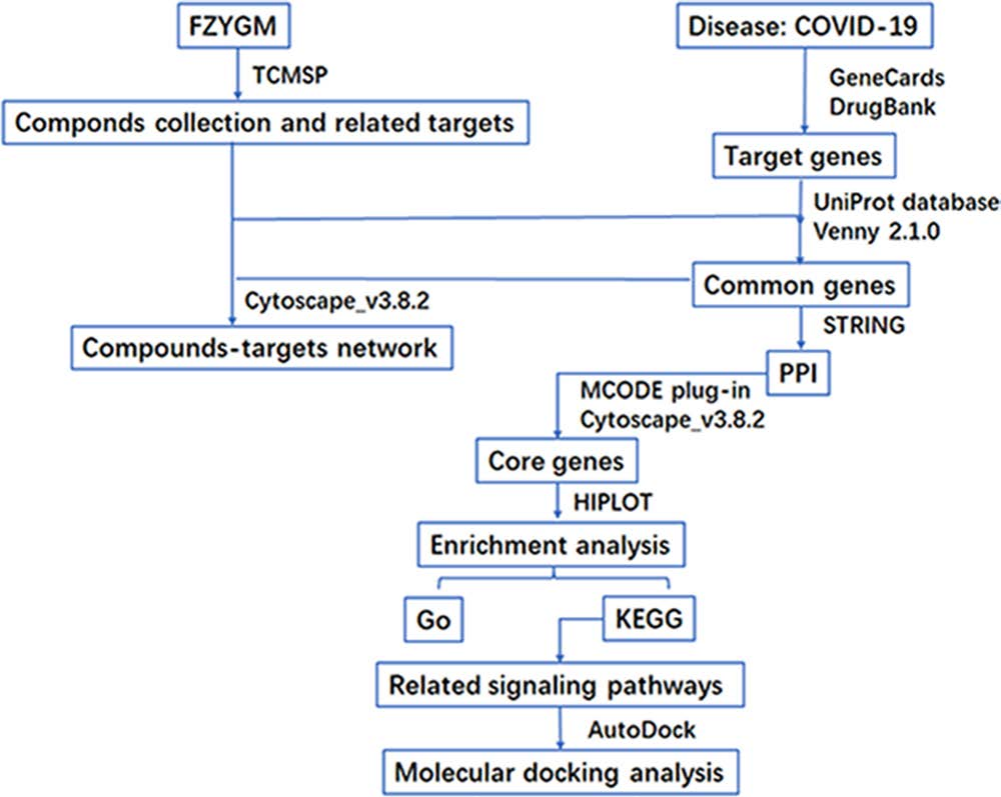
Network pharmacology analysis workflow.

Network pharmacology can integrate Chinese medicine, Chinese medicine ingredients and component targets to build the herbal-component-target network.^[12,13]^ This network involved in the analysis has laid the foundation of active ingredients and mechanism of Chinese herbal compound. Molecular docking technology can predict the binding stability between drug and receptor by predicting the interaction mode and binding energy between receptor and drug molecules, which can be used as an important tool for drug screening and design.^[14,15]^ In this study, our main objective is to explore the potential mechanism of FZYGM for the treatment of COVID-19 using molecular docking and network pharmacology approaches, providing a theoretical basis to support this mixture. We will also identify the active ingredients that play a role in the mixture. The detailed workflow diagram of the study is shown in Figure 1.

## 2. Methods

### 2.1. Establishment of disease and herbal database as well as herbal-active ingredient network

Using the traditional Chinese medicine systems pharmacology database and analysis platform (TCMSP) database (https://old.tcmsp-e.com/tcmsp.php), the active ingredients in FZYGM Chinese medicine and the target proteins corresponding to the active ingredients were screened with the oral bioavailability greater than or equal to 30%^[16–18]^ and drug-likeness greater than or equal to 0.18 as the conditions. The data filtered by the TCMSP database were processed and the relationships established between the selected herbs and their active ingredients were imported into the software Cytoscape_v3.8.2 for visualization and analysis, and a network relationship diagram between them was generated.

Search for disease-related genes in 2 major databases, GeneCards (https://www.genecards.org/) and DrugBank (https://www.drugbank.ca/), using the keyword “COVID-19.”

### 2.2. Analysis of the common targets of FZYGM and COVID-19

Using the protein database Uniprot (https://www.uniprot.org/), the gene names of the target proteins corresponding to the active ingredients of traditional Chinese medicine were searched, and the FZYGM gene database was set up to join the originally constructed database. The gene databases of FZYGM and COVID-19 were imported into Venny 2.1.0 online tool (https://bioinfogp.cnb.csic.es/tools/venny/index.html) for cross-tabulation analysis, and the common genes of FZYGM and COVID-19 were derived.

### 2.3. Construction of active ingredient-target-disease interaction network for FZYGM and COVID-19

In the established FZYGM total database, the active ingredients corresponding to the common targets of drugs and diseases were selected to establish a cross-target-active ingredient database, which was imported into the software Cytoscape_v3.8.2 for visualization and analysis to derive the active ingredient-target-disease interaction network.

### 2.4. Construction of protein-protein interactions (PPI) network maps

STRING (https://www.string-db.org/) is a database for building functional protein association networks. Select the function module “Multiple Proteins,” import the cross-targets of FZYGM and COVID-19, select the organisms “Homo sapiens,” and derive the PPI network of common targets. Export the PPI network to a “tsv” file.

### 2.5. Construction of core network

From the “tsv” file exported from STRING, select the 20 targets with the highest number of corresponding nodes and import them into an Excel worksheet to create a circle diagram. Import the “tsv” file into Cytoscape_v3.8.2 for PPI network visualization. MCODE plug-in is used to analyze the sub-network of PPI network, and the network with the highest score is selected as the core network of this study for in-depth analysis.

### 2.6. Gene ontology (GO) and Kyoto encyclopedia of genes and genomes (KEGG) pathway enrichment analysis

In order to reflect the biological processes (BP), molecular functions, cell composition (CC) and pathways of FZYGM in the treatment of COVID-19, the core genes were introduced into the visualization-driven bioinformatics analysis online website Hiplot (https://hiplot.com.cn) for GO and KEGG pathway enrichment analysis, from which bubble charts or histograms can be drawn for visual analysis. Select the latest KEGG database, set *P* < 0.01 and the size of the final output image, and choose the top 20 results with the highest enrichment. Finally export the “xlsx” file and picture file. Hiplot, as a visualization-based statistical analysis tool, is essentially a simplified use of the R language. The R package “clusterProfiler” is applied in the enrichment tool we use. In the histogram, the smaller the *P* value, the higher the degree of enrichment; the longer the bar, the more enriched genes.

### 2.7. Molecular docking

After selecting the required signaling pathways from the KEGG enriched signaling pathways, the gene IDs enriched in each signaling pathway were queried in the exported “xlsx” worksheet, then retrieved and converted to gene names using the KEGG database (https://www.kegg.jp/) to create signaling pathway database. The database was imported into Cytoscape_v3.8.2 for visual analysis. In the established FZYGM database, the core genes enriched by signaling pathway were searched and the corresponding active ingredients were found to establish the relation table for molecular docking.

Molecular docking can explain the interaction mechanism and binding activity of the active component with the target protein to some extent. The structure of the compound was downloaded from the Pubchem database (https://pubchem.ncbi.nlm.nih.gov/) in SDF format and then converted from SDF to MOL2 format using Chem 3D software. The protein structure was downloaded from the RCSB database in the PDB format. PyMOL software was used to modify the protein molecules obtained from the PDB database, and the “remove waters” command was used to remove the water of crystallization from the protein structure. At the same time, use the “GetBox” plug-in, select the ligand to obtain the coordinates of the active pocket at the time of docking, use the “remove” command to remove the ligand, and then export the protein molecule in pdb. format to obtain the original ligand of the target protein. The original ligand of the target protein can also be obtained. Using the “Edit” module of the GUI tool in AutoDock Tools, each protein is assigned a polar hydrogen atom, a charge, and an atom type, while the “Torsion Tree” is used to determine. After completion, the target proteins and small molecules are exported in pdbqt. format for molecular docking. The target proteins and small molecules in pdbqt. format obtained after pretreatment were used as objects for molecular docking using AutoDock Vina software. Each molecule was docked from 10 conformational angles, and the binding free energy resulting from the receptor-ligand interaction obtained in each orientation during docking was calculated to determine the optimal conformation of the complex. Finally, Discovery Studio 2020 software was used for the constitutive relationship analysis.

## 3. Results

### 3.1. Network of herbs and active ingredients in FZYGM

**Figure 2.**
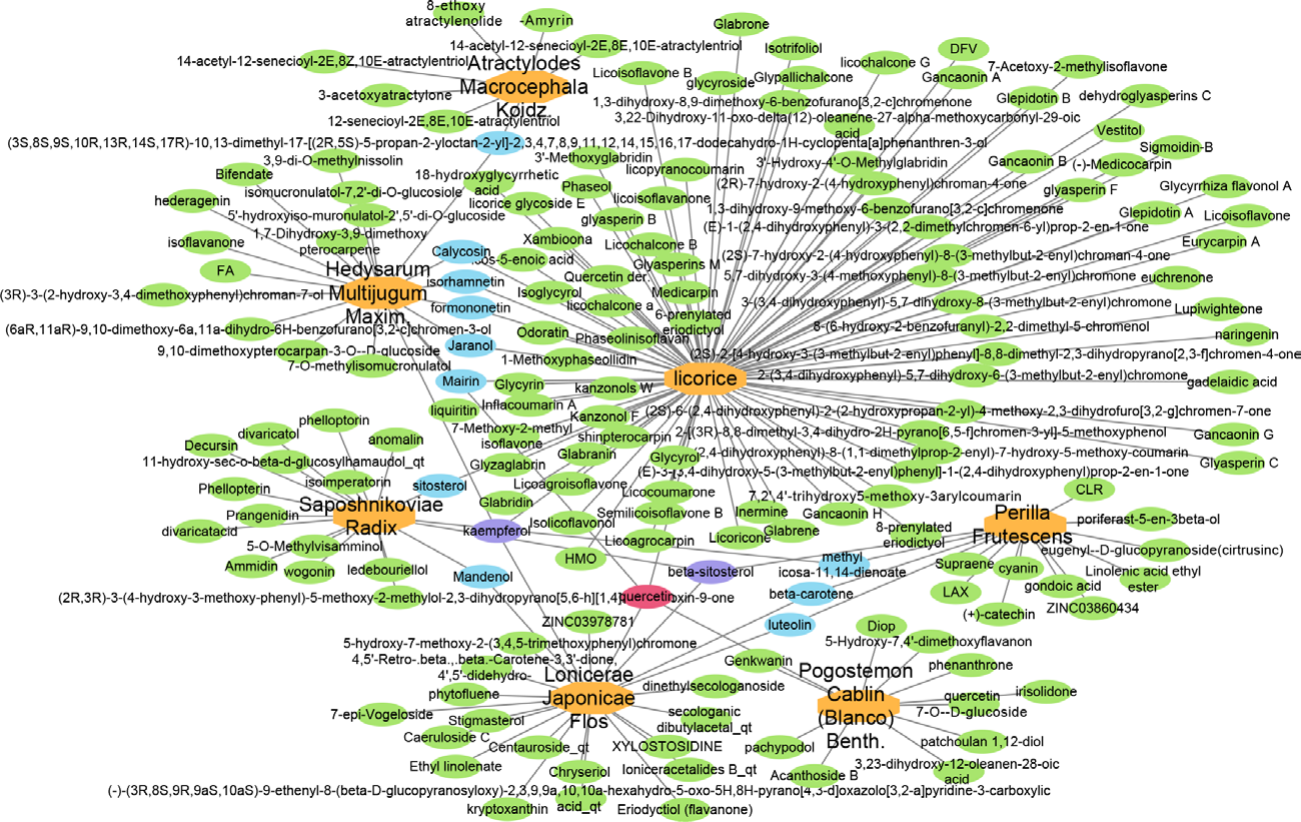
FZYGM active ingredient and Chinese herbal medicine network diagram. The yellow circular nodes represent herbal medicines, and the other color circular nodes represent the active ingredients corresponding to the pairs of herbal medicines. Among them, green nodes correspond to one herbal medicine, blue correspond to 2, purple correspond to 3, and red correspond to 4. FZYGM = Fuzheng Yugan Mixture.

Based on the ADME model, we screened 7 herbal medicines (*Hedysarum Multijugum Maxim, Saposhnikoviae Radix, Atractylodes Macrocephala Koidz, licorice, Lonicerae Japonicae Flos, Pogostemon Cablin (Blanco*) *Benth* and *Perilla Frutescens*) contained a total of 167 active ingredients. Figure 2 shows the relationship between herbs and active ingredients analyzed with Cytoscape_v3.8.2.

### 3.2. Common targets of FZYGM and COVID-19

**Figure 3.**
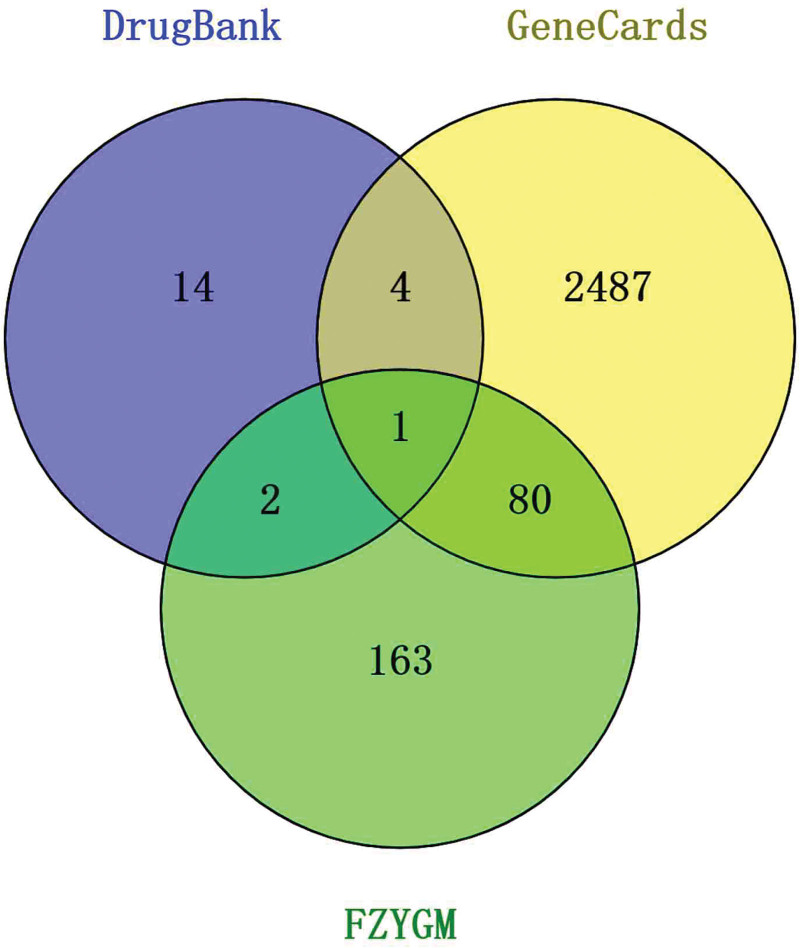
Cross-target Venn diagram of FZYGM and COVID-19. COVID-19 = coronavirus disease 2019, FZYGM = Fuzheng Yugan Mixture.

In the TCMSP database, the corresponding targets were derived from the active ingredients retrieved, totaling 246. Using COVID-19 as the keyword, the relevant genes were retrieved using GeneCards and DrugBank databases, and 2572 targets were derived from GeneCards and 21 targets from DrugBank, thus a total of 2593 relevant targets about COVID-19 were obtained. The above targets were imported into the Venny 2.1.0 online tool to create a Wayne diagram (Fig. 3), and it shows that there are 83 crossover targets for FZYGM and COVID-19.

### 3.3. Common target-active component relationship diagram of FZYGM and COVID-19

**Figure 4.**
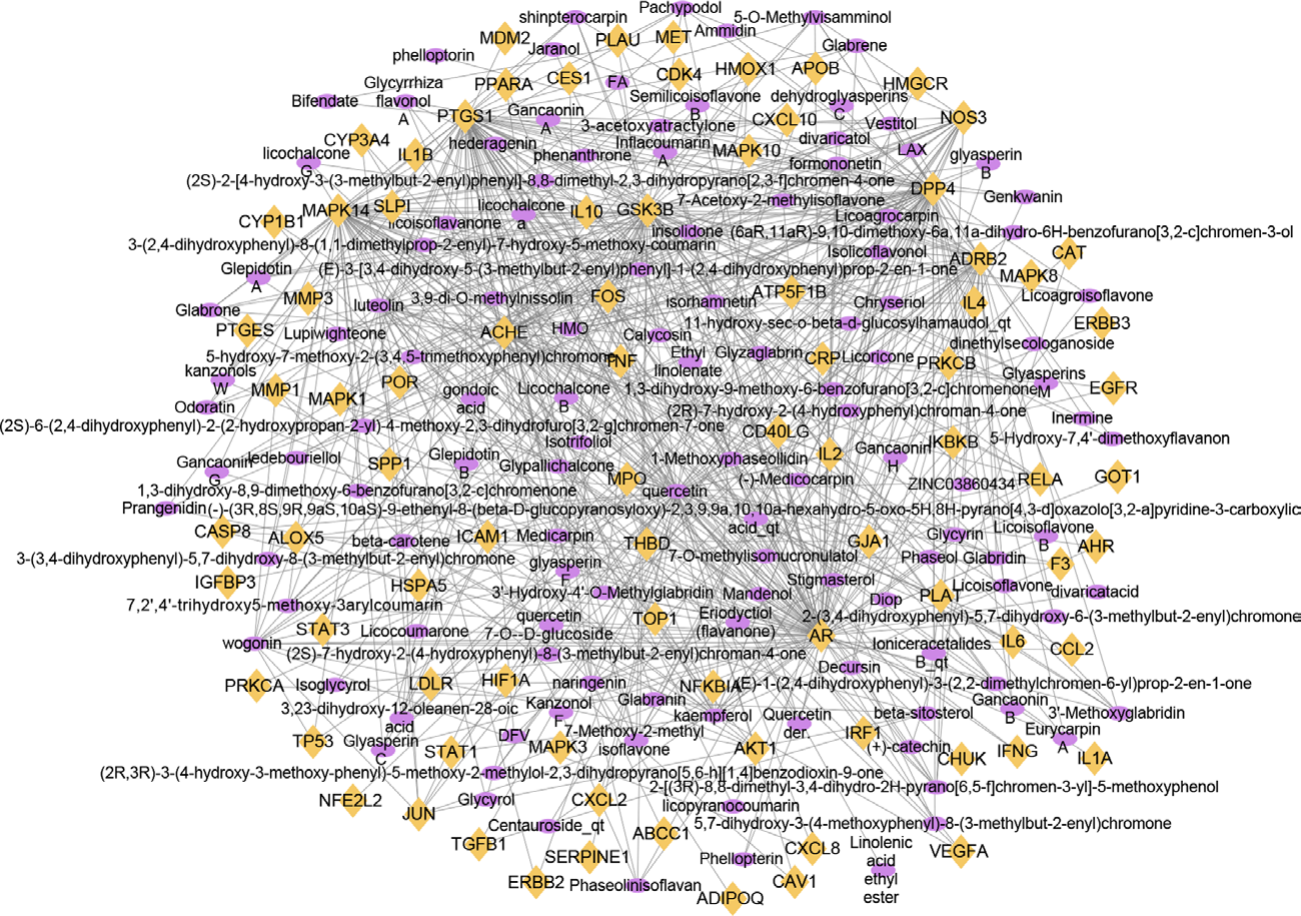
Active ingredient-target-disease interaction network of FZYGM and COVID-19. Yellow prismatic nodes represent cross-targets and purple circular nodes represent active ingredients. COVID-19 = coronavirus disease 2019, FZYGM = Fuzheng Yugan Mixture.

A total of 122 active ingredients corresponding to the common targets were searched in the established FZYGM database, and a total of 580 pairs of correspondence were found. The correspondence was imported into Cytoscape_v3.8.2 for visualization and analysis, and a common target-active ingredient relationship diagram was obtained as shown in Figure 4.

### 3.4. Core network

**Figure 5.**
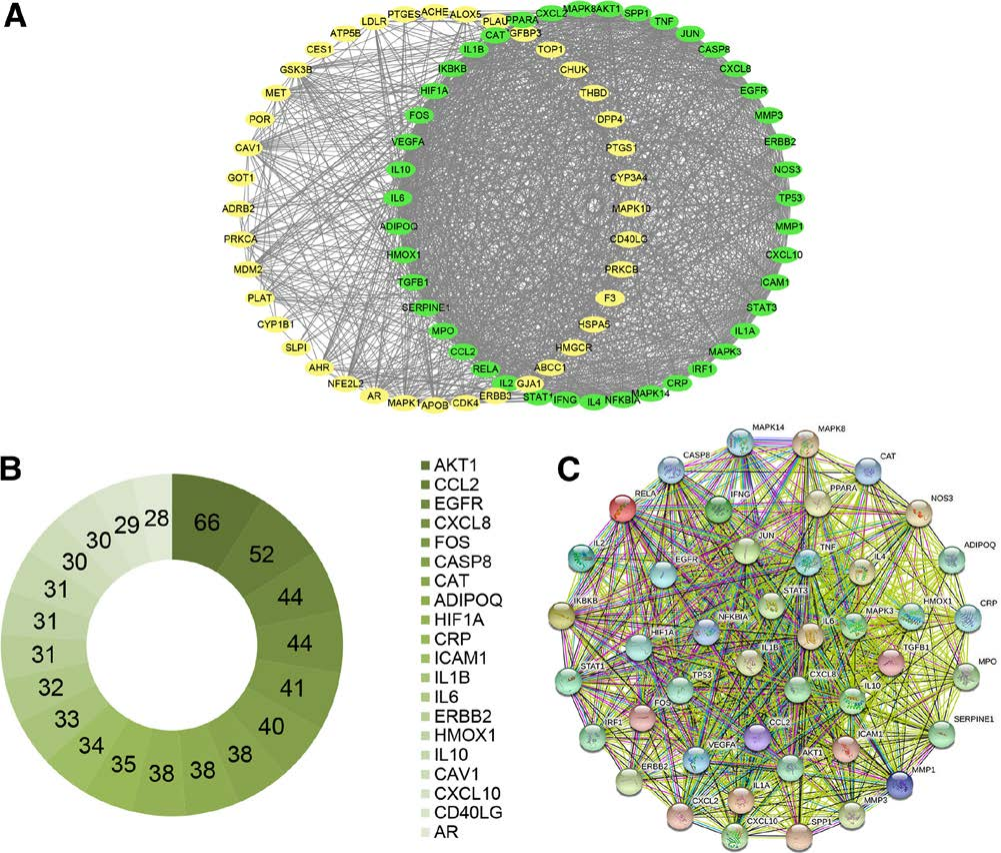
Cross-target analysis of FZYGM and COVID-19. crossover genes and core gene network of FZYGM and COVID-19: the core network is free out of the green nodes forming circles (A). Statistical results of the circle plot of the interaction between FZYGM and COVID-19 regulatory genes (B). PPI network diagram of core target interactions (C). COVID-19 = coronavirus disease 2019, FZYGM = Fuzheng Yugan Mixture, PPI = protein-protein interactions.

First, we obtained the PPI network of COVID-19 and FZYGM common targets from the STRING online database. After that, the top 20 nodes with the highest number of connected edges (e.g., AKT1, CCL2, EGFR, CXCL8) were selected to construct the circle diagram shown in Figure 5B. Finally, through the visualization of Cytoscape_v3.8.2 and the analysis of MCODE, we select the top-ranked network with a score of 36.286 as the core network, containing a total of 43 nodes and 762 edges, as shown in Figure 5A

### 3.5. PPI network analysis

The 43 core targets derived from Cytoscape_v3.8.2 analysis were imported into the STRING online database, and the PPI graph was constructed as shown in Figure 5C. The graph contains 43 nodes with 762 edges, an average node degree of 35.4, an average local clustering coefficient of 0.889, an expected number of edges of 194, and a PPI enrichment *P*-value < 1.0e-16.

### 3.6. GO and KEGG pathway enrichment analysis

**Figure 6.**
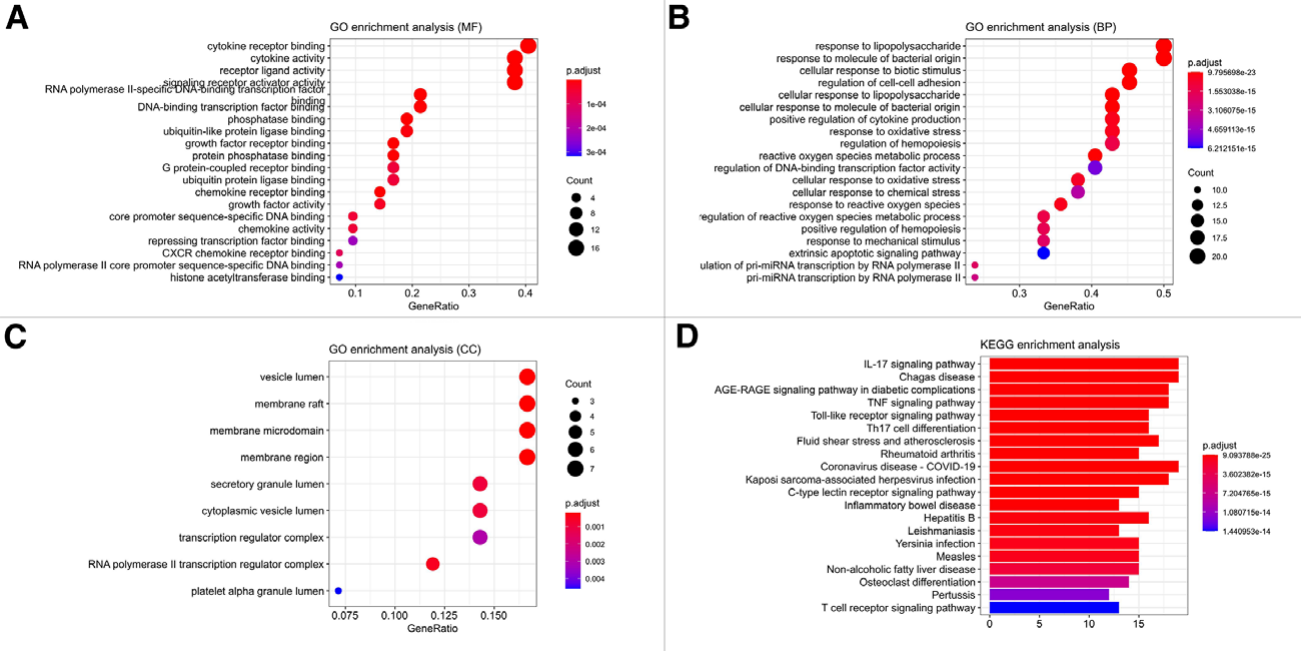
GO and KEGG pathway enrichment analysis graphs. The results of GO enrichment are MF (A), BP (B), and CC (C), where the horizontal coordinate is GeneRatio, while the bigger bubbles represent the higher number of contained genes. The results of KEGG pathway enrichment are (D), where the horizontal coordinate is the number of enriched genes and the vertical coordinate is the name of the enriched pathway. The smaller *P* value in these plots represents a higher enrichment level, and also a redder color. BP = biological processes, CC = cell composition, GO = gene ontology, KEGG = Kyoto encyclopedia of genes and genomes, MF = molecular functions.

The results of GO enrichment can be seen in Figure 6, which shows that the 43 core targets mainly act on receptor proteins in the process of cytokines, chemokines and signaling pathways, as well as some protein phosphatases, etc (Fig. 6A). The BP involved are mainly focused on the response to molecules of bacterial origin, the response to lipopolysaccharide and the metabolism of reactive oxygen species (Fig. 6B). The CC process shows that most of the targets effect on vesicles, cell membrane systems, RNA polymerase II transcriptional regulatory complexes and platelet alpha particles (Fig. 6C). A total of 185 signaling pathways were enriched by KEGG, and the top 20 were selected to be displayed as bar graphs (Fig. 6D).

### 3.7. Validation results of the interaction of active ingredients with target genes

**Figure 7.**
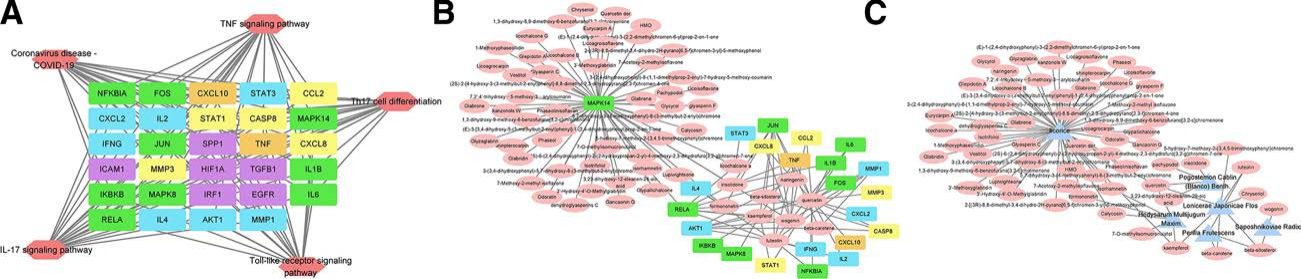
Key signaling pathways and target genes of FZYGM and COVID-19 crossover genes. Purple rectangular nodes represent target genes contained in one signaling pathway, blue rectangular nodes contain in 2 signaling pathways, yellow rectangular nodes contain in 3 signaling pathways, orange rectangular nodes contain in 4 signaling pathways, green rectangular nodes contain in 5 signaling pathways; red hexagonal nodes represent signaling (A). The key target genes and ingredients of FZYGM and COVID-19 crossover genes: the pink circular nodes represent the active ingredients of Chinese medicine corresponding to the targets; the rectangular nodes correspond to the above Fig (B). Key ingredients and herbs of FZYGM and COVID-19 crossover genes: blue triangle nodes represent herbs corresponding to the active ingredients; pink circle nodes correspond to above (C). COVID-19 = coronavirus disease 2019, FZYGM = Fuzheng Yugan Mixture.

Among the top 20 signaling pathways enriched by KEGG, interleukin (IL)-17 signaling pathway, tumor necrosis factor (TNF) signaling pathway, toll-like receptor (TLR) signaling pathway, Th17 cell differentiation, and Coronavirus disease - COVID-19 5 signaling pathways were analyzed, which included 29 core genes. Then the corresponding active ingredients were collected from the FZYGM database, and 60 active ingredients related to them were found, and there were 6 kinds of related traditional Chinese medicines. These results were imported into Cytoscape_v3.8.2 for visualization and analysis, and the results were shown in Figure 7.

**Figure 8.**
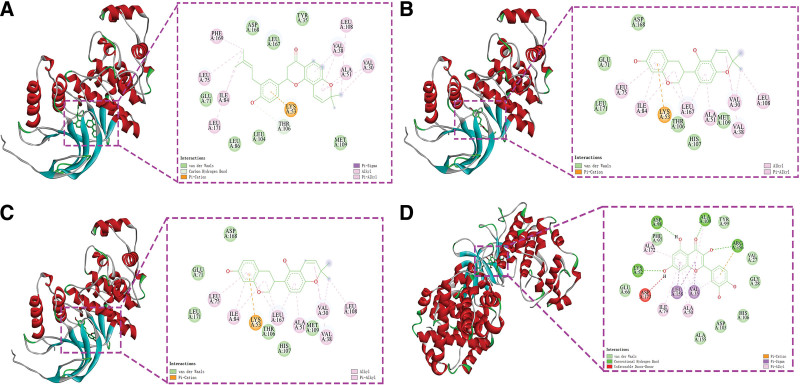
The best combination visual representation of molecular docking. These 4 groups are: (2S)-2-[4-hydroxy-3-(3-methylbut-2-enyl)phenyl]-8,8-dimethyl-2,3-dihydropyrano[2,3-f]chromen-4-one acts on MAPK14 (A), phaseolinisoflavan acts on MAPK14 (B), Glabrene acts on MAPK14 (C), quercetin acts on STAT1 (D). MAPKs = mitogen-activated protein kinases.

The 29 core genes were molecularly docked with their corresponding active ingredients to verify the effects of the active ingredients, and the docking results are shown in Table 1. Generally speaking, a binding energy of less than −5 kcal/mol indicates that the 2 molecules are firmly bound. The top 4 groups with the best docking are shown separately in Figure 8.

**Table 1:** The combination of the best docking model energy.

Molecule name	Target proteins	PDB ID	Binding Energy (kcal/mol)
(2S)-2-[4-hydroxy-3-(3-methylbut-2-enyl)phenyl]-8,8-dimethyl-2,3-dihydropyrano[2,3-f]chromen-4-one	MAPK14	3DT1	−10.2
Phaseolinisoflavan	MAPK14	3DT1	−9.9
Glabrene	MAPK14	3DT1	−9.9
quercetin	STAT1	6QTJ	−9.9
shinpterocarpin	MAPK14	3DT1	−9.8
irisolidone	RELA	3QXY	−9.7
kaempferol	STAT1	6QTJ	−9.6
Lupiwighteone	MAPK14	3DT1	−9.4
kanzonols W	MAPK14	3DT1	−9.4
2-[(3R)-8,8-dimethyl-3,4-dihydro-2H-pyrano[6,5-f]chromen-3-yl]-5-methoxyphenol	MAPK14	3DT1	−9.4
kaempferol	RELA	3QXY	−9.4
kaempferol	IKBKB	4KIK	−9.3
licochalcone a	RELA	3QXY	−9.3
beta-carotene	AKT1	4GV1	−9.3
Glabridin	MAPK14	3DT1	−9.2
quercetin	RELA	3QXY	−9.2
(2S)-6-(2,4-dihydroxyphenyl)-2-(2-hydroxypropan-2-yl)-4-methoxy-2,3-dihydrofuro[3,2-g]chromen-7-one	MAPK14	3DT1	−9.1
Licoagroisoflavone	MAPK14	3DT1	−9.1
quercetin	MMP3	1HY7	−9
beta-sitosterol	JUN	2G01	−9
Glabrone	MAPK14	3DT1	−9
isorhamnetin	RELA	3QXY	−9
wogonin	RELA	3QXY	−9
luteolin	RELA	3QXY	−9
formononetin	JUN	2G01	−8.9
luteolin	JUN	2G01	−8.9
Phaseol	MAPK14	3DT1	−8.9
irisolidone	JUN	2G01	−8.8
3-(3,4-dihydroxyphenyl)-5,7-dihydroxy-8-(3-methylbut-2-enyl)chromone	MAPK14	3DT1	−8.8
Licoisoflavone	MAPK14	3DT1	−8.8
Eurycarpin A	MAPK14	3DT1	−8.8
3’-Methoxyglabridin	MAPK14	3DT1	−8.8
dehydroglyasperins C	MAPK14	3DT1	−8.8
glyasperin F	MAPK14	3DT1	−8.7
(E)-1-(2,4-dihydroxyphenyl)-3-(2,2-dimethylchromen-6-yl)prop-2-en-1-one	MAPK14	3DT1	−8.7
Glyzaglabrin	MAPK14	3DT1	−8.7
3’-Hydroxy-4’-O-Methylglabridin	MAPK14	3DT1	−8.7
naringenin	RELA	3QXY	−8.7
kaempferol	MAPK8	2XRW	−8.6
kaempferol	JUN	2G01	−8.6
wogonin	JUN	2G01	−8.6
isorhamnetin	MAPK14	3DT1	−8.6
wogonin	MAPK14	3DT1	−8.6
Glyasperin C	MAPK14	3DT1	−8.6
Glepidotin A	MAPK14	3DT1	−8.6
Licoagrocarpin	MAPK14	3DT1	−8.6
Chryseriol	MAPK14	3DT1	−8.6
Pachypodol	MAPK14	3DT1	−8.6
quercetin	JUN	2G01	−8.5
(E)-3-[3,4-dihydroxy-5-(3-methylbut-2-enyl)phenyl]-1-(2,4-dihydroxyphenyl)prop-2-en-1-one	MAPK14	3DT1	−8.5
1-Methoxyphaseollidin	MAPK14	3DT1	−8.5
quercetin	FOS	5PAM	−8.4
7-Methoxy-2-methyl isoflavone	MAPK14	3DT1	−8.4
5,7-dihydroxy-3-(4-methoxyphenyl)-8-(3-methylbut-2-enyl)chromone	MAPK14	3DT1	−8.4
7-Acetoxy-2-methylisoflavone	MAPK14	3DT1	−8.4
Vestitol	MAPK14	3DT1	−8.4
3,23-dihydroxy-12-oleanen-28-oic acid	MAPK14	3DT1	−8.4
quercetin	MAPK14	3DT1	−8.4
quercetin	AKT1	4GV1	−8.4
beta-sitosterol	CASP8	4PS1	−8.3
beta-carotene	JUN	2G01	−8.3
Glycyrol	MAPK14	3DT1	−8.3
naringenin	AKT1	4GV1	−8.3
Quercetin der.	MAPK14	3DT1	−8.2
Gancaonin G	MAPK14	3DT1	−8.2
5-hydroxy-7-methoxy-2-(3,4,5-trimethoxyphenyl)chromone	MAPK14	3DT1	−8.2
luteolin	MAPK14	3DT1	−8.2
luteolin	AKT1	4GV1	−8.2
formononetin	MAPK14	3DT1	−8.1
3-(2,4-dihydroxyphenyl)-8-(1,1-dimethylprop-2-enyl)-7-hydroxy-5-methoxy-coumarin	MAPK14	3DT1	−8.1
7,2’,4’-trihydroxy-5-methoxy-3-arylcoumarin	MAPK14	3DT1	−8.1
kaempferol	AKT1	4GV1	−8.1
quercetin	MMP1	1CGE	−8.1
Glypallichalcone	MAPK14	3DT1	−8
licochalcone G	MAPK14	3DT1	−8
1,3-dihydroxy-9-methoxy-6-benzofurano[3,2-c]chromenone	MAPK14	3DT1	−8
HMO	MAPK14	3DT1	−8
quercetin	IFNG	1FYH	−8
luteolin	IFNG	1FYH	−8
luteolin	TNF	2AZ5	−7.9
7-O-methylisomucronulatol	MAPK14	3DT1	−7.8
Calycosin	MAPK14	3DT1	−7.8
Isotrifoliol	MAPK14	3DT1	−7.8
Licochalcone B	MAPK14	3DT1	−7.8
1,3-dihydroxy-8,9-dimethoxy-6-benzofurano[3,2-c]chromenone	MAPK14	3DT1	−7.8
licochalcone a	MAPK14	3DT1	−7.8
Odoratin	MAPK14	3DT1	−7.8
irisolidone	MAPK14	3DT1	−7.8
luteolin	MMP1	1CGE	−7.8
kaempferol	TNF	2AZ5	−7.7
quercetin	TNF	2AZ5	−7.7
wogonin	TNF	2AZ5	−7.7
wogonin	AKT1	4GV1	−7.7
irisolidone	TNF	2AZ5	−7.6
licochalcone a	STAT3	5AX3	−7.6
quercetin	IL1B	5R85	−7.2
wogonin	IL6	1N26	−7.2
kaempferol	MMP1	1CGE	−7.2
beta-carotene	CASP8	4PS1	−7
quercetin	IL6	1N26	−7
quercetin	CASP8	4PS1	−6.8
irisolidone	IL1B	5R85	−6.8
luteolin	IL6	1N26	−6.8
wogonin	MMP1	1CGE	−6.7
quercetin	CXCL2	5OB5	−6.6
beta-carotene	MMP1	1CGE	−6.5
luteolin	IL2	1M48	−6.5
wogonin	CXCL8	6WZM	−6.3
quercetin	IL2	1M48	−6.3
quercetin	CXCL8	6WZM	−6.2
luteolin	IL4	2B8U	−6.2
quercetin	CCL2	1DOK	−5.9
quercetin	CXCL10	1O80	−5.9

IL = interleukin, TNF = tumor necrosis factor.

We found that MAPK14 showed good stability in binding to other proteins, including (2S)-2-[4-hydroxy-3-(3-methylbut-2-enyl)phenyl]-8,8-dimethyl-2,3-dihydropyrano[2,3-f]chromen-4-one with MAPK14 at a binding energy of −10.2 kcal/mol, forming hydrogen bonds via Thr106 and hydrophobic interactions with Phe169, Leu75, Leu171, Ile84, Val38, Leu108, Ala51, and Val30 (Fig. 8A); Phaseolinisoflavan with MAPK14 at an binding energy of −9.9 kcal/mol and formed hydrophobic interactions with Leu75, Ile84, Leu167, Ala51, Val30, Val38, and Leu108 (Fig. 8B); Glabrene had a binding energy of −9.9 kcal/mol with MAPK14, formed hydrogen bonding with Lys53 and Met109, and formed hydrophobic interactions with Leu167, Val38 Ala51, Ile84, and Leu75 (Fig. 8C); and lastly, quercetin had binding energy of −9.9 kcal/mol with STAT1, forming hydrogen bonds with Lys52, Asp98, Ala100, and Arg356, and hydrophobic interactions with Ala172, Ile79, Ala50, Val35, and Ala100 (Fig. 8D).

## 4. Discussion

COVID-19 is highly contagious and can cause severe lung inflammation and complications after infection, while the host has a limited immune response to viral infection and requires strong external intervention.^[19]^ To reduce the global public health damage caused by this disease, it has now gained widespread recognition that the development of effective vaccine-based prevention strategies^[20]^ and therapeutic strategies, as well as drug development, are critical in the fight against COVID-19. The treatment options for COVID-19 are diverse, including antiviral, anti-inflammatory cytokine, anti-infective, and immunotherapy,^[21]^ but there are still no effective drugs to treat this disease.^[22]^ According to the Chinese TCM Administration, the total effective rate of TCM involvement in the treatment of neocrown pneumonia has reached 90% in many regions of China. After the treatment of TCM prescriptions in the treatment, patients have significant improvements in clinical symptoms and lung imaging, indicating the great advantage of TCM for the treatment of COVID-19.^[23,24]^ However, the specific mechanism of Chinese herbal medicine adjuvant therapy for COVID-19 is still to discover.

Network pharmacology has been successfully applied to biological mechanisms studies of several herbal mixturetions^[25,26]^ and ingredients.^[27,28]^ Therefore, we employed network pharmacology approach to obtain the biological mechanisms of FZYGM at a systemic level. In the network pharmacology analysis, 246 targets related to candidate compounds and 2593 targets related to COVID-19 were identified by ADME model (oral bioavailability ≥ 30%, drug-likeness ≥ 0.18) screening, and a total of 83 cross-targets were obtained by PPI network (Fig. 3). Then, in the analysis of the core network, we obtained the core network containing 43 nodes and the top 20 Hubgenes (Fig. 5). Among the Hubgenes, AKT1 was the first one with the tightest connection, which may mediate the level of inflammatory factors in COVID-19. Current studies suggest that the exploration of the role of AKT1 in neocrown pneumonia has attracted attention.^[29–31]^ We subjected the first-ranked core network to PPI analysis again, and the results showed that its nodes have good interaction efficacy, which facilitates the next enrichment analysis.

GO enrichment analysis has revealed that multiple targets are associated with cytokine receptor binding in molecular functions and are involved in response to lipopolysaccharide, cell-cell adhesion, apoptotic signaling pathways, and oxidative stress in BP, which are localized to the vesicular lumen, membrane rafts, and membrane microstructure domains in CC. In contrast, it was shown in previous studies that SARS-CoV-2 stinging proteins could bind to lipopolysaccharides and promote inflammatory effects during infection.^[32]^ Meanwhile, in an in vitro experiment, SARS-CoV-2 was found to cause apoptosis by inducing apoptotic gene expression in human artificial pluripotent stem cell-derived brain tissue neuronal cells.^[33]^ Thus, the GO enrichment can be of value to help uncover more potential molecular mechanisms and specific sites of action of FZYGM for COVID-19 treatment.

Analysis of KEGG pathway enrichment results combined with previous studies suggested that the IL-17 signaling pathway and TLR signaling pathway may be critical in this core network. In addition, the differentiation of TNF and Th17 cells in the arthropathology of neocoronary pneumonia was examined.^[34,35]^ IL-17 is a pro-inflammatory cytokine produced mainly by Th17 cells and has received much attention for its pro-inflammatory role in diseases including infections and autoimmune diseases.^[36]^ IL-17 binds to its receptor and exerts its biological through MAPK and NF-kB pathways role through MAPK and NF-kB pathways.^[37,38]^ As assessed in patients with COVID-19, excessive activation of Th17 cells leads to increased production of IL-17 cytokines. Subsequently, the increase in IL-17 leads to severe tissue damage and may trigger pulmonary edema through cytokine storm in pulmonary viral infections. A retrospective study showed that targeted inhibition of IL-17 and thus prevention of lung injury is a promising therapeutic strategy for COVID-19.^[39]^ TLR is an essential protein molecule involved in the immune response, collaborating with various other receptors in the recognition of viral particles and in the regulation of immunity.^[40,41]^ Activation of the TLR signaling pathway induces the expression of various pro-inflammatory factors and type I interferons that induce antiviral responses.^[42]^ Studies have shown that The TLR3- and TLR7-dependent production of type I IFNs is an important mechanism for the body’s resistance to SARS-CoV-2, while innate TLR3- and IRF7-dependent type I IFN immune errors may induce severe COVID-19 infection.^[43,44]^ Therefore, TLRs may be potential targets for manufacturing SARS-CoV-2 vaccines and targeted drugs.^[45]^

To validate the KEGG enrichment results, we molecularly docked the core targets in the above 5 signaling pathways with the corresponding Chinese herbal active ingredients.^[46]^ Molecular docking showed that (2S)-2-[4-hydroxy-3-(3-methylbut-2-enyl) phenyl]-8,8-dimethyl-2,3-dihydropyrano[2,3-f] chromen-4-one, Phaseolinisoflavan, Glabrene, quercetin, shinpterocarpin, irisolidone and kaempferol have good binding activity with MAPK14, STAT1 and RELA targets (Fig. 8). Coronaviruses have been proved to involve 3 P38 mitogen-activated protein kinases (MAPKs), the Jun amino-terminal kinases and extracellular signal-regulated kinases) pathways for viral pathogenesis.^[32,47]^ MAPKs mediated cross-talk activation of transcription factor family nuclear factor kappa-B (NF-κB) was reported by Saccani et al^[48]^ MAPK14 is one of the 4 p38 MAPKs which confirm a crucial role in the cascades of cellular reactions induced by an extracellular stimulus such as proinflammatory cytokines. MAPK14 pathway regulates the translational level of TNF which can further stimulate NF-κB non-canonically.^[49,50]^ Hence, increased activation of MAPK14 and cross-talk activation of NF-κB pathway might be the reason for the thrombosis, inflammation and vasoconstriction in COVID-19.^[51]^ Anomalous upregulation of STAT1 is found in B and T cells and monocytes from mild and severe COVID-19 patients compared to controls, While the phosphorylation of STAT1 is enhanced in severely affected COVID-19 patients.^[52]^ RELA, also named p65, is an important member of the NF-κB that play an essential role in inflammation,^[53–55]^ while excessive activation of the NF-κB pathway has been thought to be associated with the pathogenesis of the severe/critical COVID-19 phenotype.^[56]^

We revealed the pharmacological targets and signaling pathways of FZYGM for the treatment of COVID-19 by using network pharmacology and molecular docking techniques. Our results indicate that FZYGM can act at multiple cellular sites and participate in multiple biochemical responses, acting through mechanisms of inflammation, cell necrosis, and immune-related signaling pathways. Based on the validation results of molecular docking, we also know that the pharmacological effects of FZYGM in treating COVID-19 may be achieved by modulating the core targets of its related signaling pathways. These results suggest that FZYGM may exert anti-inflammatory effects through multiple pathways to treat COVID-19. It should be noted that due to the limitations of computer prediction methods, network pharmacology and molecular docking are only a preliminary exploration of the potential mechanism of drug action, and further experimental studies are still needed to validate it subsequently. However, due to the limitation of the number of patients, the therapeutic effect for COVID-19 still needs to be confirmed in future clinical work.

## 5. Conclusion

In this study, network pharmacology and molecular docking were used to harvest the pivotal biotargets and potential therapeutic mechanisms of FZYGM in the treatment of COVID-19. Our results indicate that FZYGM can achieve intervention of COVID-19 by regulating the level of inflammatory cytokines and immunomodulation. Molecular docking showed that FZYGM could initiatively bind to the receptor and had strong binding energy, suggesting multi-dimensional mechanism. We also identified active ingredients (2S)-2-[4-hydroxy-3-(3-methylbut-2-enyl) phenyl]-8,8-dimethyl-2,3-dihydropyrano[2,3-f] chromen-4-one, Phaseolinisoflavan, Glabrene, quercetin, shinpterocarpin, irisolidone and kaempferol in FZYGM that may play a key role in the action of FZYGM on COVID-19. Conclusion of this study provides better support for subsequent experiments and pharmacological mechanism in the treatment of COVID-19.

## Acknowledgments

The authors are thankful to Tongde Hospital of Zhejiang Province for providing assistance with the pharmaceutical mixturetion of this study. At the same time, particular thanks to Hua Yan from Wenzhou University for her help in improving the language quality of this article.

## Author contributions

**Conceptualization:** Jie Zhou, Tianyue Wang.

**Funding acquisition:** Jie Zhou.

**Methodology:** Tianyue Wang.

**Project administration:** Jie Zhou.

**Software:** Xinyu Jiang.

**Supervision:** Tianyue Wang.

**Validation:** Zhongming Yu, Xueya Gu.

**Visualization:** Xinyu Jiang, Yanmin Ruan.

**Writing – original draft:** Xinyu Jiang, Jie Zhou, Tianyue Wang.

**Writing – review & editing:** Jie Zhou, Ying Lu, Tianyue Wang.
